# Rescue of Murine F508del CFTR Activity in Native Intestine by Low Temperature and Proteasome Inhibitors

**DOI:** 10.1371/journal.pone.0052070

**Published:** 2012-12-21

**Authors:** Martina Wilke, Alice Bot, Huub Jorna, Bob J. Scholte, Hugo R. de Jonge

**Affiliations:** 1 Department of Clinical Genetics, Erasmus Medical Center, Rotterdam, The Netherlands; 2 Department of Biochemistry, Erasmus Medical Center, Rotterdam, The Netherlands; 3 Erasmus Laboratory Animal Science Center, Erasmus Medical Center, Rotterdam, The Netherlands; 4 Department of Cell Biology, Erasmus Medical Center, Rotterdam, The Netherlands; 5 Department of Gastroenterology & Hepatology, Erasmus Medical Center, Rotterdam, The Netherlands; Johns Hopkins School of Medicine, United States of America

## Abstract

Most patients with Cystic Fibrosis (CF) carry at least one allele with the F508del mutation, resulting in a CFTR chloride channel protein with a processing, gating and stability defect, but with substantial residual activity when correctly sorted to the apical membranes of epithelial cells. New therapies are therefore aimed at improving the folding and trafficking of F508del CFTR, (CFTR correctors) or at enhancing the open probability of the CFTR chloride channel (CFTR potentiators). Preventing premature breakdown of F508del CFTR is an alternative or additional strategy, which is investigated in this study. We established an ex vivo assay for murine F508del CFTR rescue in native intestinal epithelium that can be used as a pre-clinical test for candidate therapeutics. Overnight incubation of muscle stripped ileum in modified William's E medium at low temperature (26°C), and 4 h or 6 h incubation at 37°C with different proteasome inhibitors (PI: ALLN, MG-132, epoxomicin, PS341/bortezomib) resulted in fifty to hundred percent respectively of the wild type CFTR mediated chloride secretion (forskolin induced short-circuit current). The functional rescue was accompanied by enhanced expression of the murine F508del CFTR protein at the apical surface of intestinal crypts and a gain in the amount of complex-glycosylated CFTR (band C) up to 20% of WT levels. Sustained rescue in the presence of brefeldin A shows the involvement of a post-Golgi compartment in murine F508del CFTR degradation, as was shown earlier for its human counterpart. Our data show that proteasome inhibitors are promising candidate compounds for improving rescue of human F508del CFTR function, in combination with available correctors and potentiators.

## Introduction

The Cystic Fibrosis Transmembrane Conductance Regulator (CFTR), a cAMP- and cGMP-activated chloride channel, is expressed in the apical membrane of various epithelia, including airway, intestine, and exocrine glands [1], [2], [3]. Mutations in the CFTR encoding gene cause the lethal autosomal recessive disorder cystic fibrosis (CF). Currently there are more then 1800 mutations identified in the CFTR gene (available at http://www.genet.sickkids.on.ca/ or http://www.cftr2.org (includes clinical information)), but a single mutation (F508del) is present on at least one allele in 90% of the CF patients [4]. Both the human and the mouse orthologs of F508del CFTR are temperature-sensitive folding and trafficking mutants [5], [6]. The mutant CFTR protein is retained in the endoplasmatic reticulum (ER) through prolonged association with molecular chaperones, ubiquitinated and retrotranslocated into the cytosol, and finally degraded by the ubiquitin (Ub)-proteasome pathway as part of ER-associated degradation (ERAD) [7], [8], [9], [10]. In most cultured cell models and native epithelia, a small portion of the F508del protein can escape the quality control (QC) system of the ER, and subsequently undergo complex glycosylation in the Golgi compartment and transfer to the apical membrane of epithelial cells. The F508del protein at the cell surface is active as a chloride channel, though with a strongly reduced open probability and considerably higher turnover rate as compared to wild type CFTR [8], [11], [12], [13]. The instability of rescued F508del CFTR was attributed to unfolding and subsequent ubiquitination, endocytosis, and lysosomal degradation by a peripheral protein QC system sharing multiple chaperones and co-chaperones (e.g. UbcH5; CHIP; Hsp70/90) with the QC in the ER [14]. Attempts to correct the F508del *CFTR* allele-specific phenotype are currently focussed on the selection of compounds that overcome the inefficient folding of the mutant protein (correctors), or enhance the CFTR chloride channel activity (potentiators) [15], [16], [17]. Small molecule correctors may also act as pharmacological chaperones and enhance the cell-surface stability of F508del-CFTR [13]. Partial rescue of the human F508del CFTR protein has been demonstrated in cell culture using different strategies. Initially, restoration of F508del CFTR processing was accomplished by low temperature incubation [18], [19]. Subsequently, competition with truncated CFTR constructs [20], [21], chemical chaperones [22], [23], transcriptional regulators [24], pharmacological chaperones (e.g. MPB, miglustat [25], second-site aminoacid substitutions [26] or deletion of the regulatory insertion in nucleotide binding domain 1 of CFTR [27] proved to be effective. Numerous F508del potentiators and correctors have been identified by either high throughput screening (HTS) or modification(s) of available lead compounds [28], [29], [30], [31], [32], [33]. Most of these studies have been performed with primary or immortalised human airway cells in vitro. Recent studies showed that the relative efficacy of different types of correctors depends on the cell type and experimental context [29], [34]. This limits the predictive value of in vitro data for clinical applications assays and stresses the importance of choosing models that reflect the *in vivo* situation. Prior to expensive and potentially harmful clinical trials, testing of a promising candidate in the context of a fully differentiated organ is advisable. In this study we have used the F508del (*Cftr^tm1Eur^*) mouse strain generated at the Erasmus MC Rotterdam for this purpose [3], [6], [35]. In this model, made by ‘hit and run’ homologous recombination, a F508del mutant form of Cftr mRNA is present at normal expression levels in all affected organs. To avoid complications related to unfavourable pharmacokinetics of candidate correctors or potentiators that may obscure effects on mutant CFTR activity during *in vivo* application, a major effort in our laboratory has been to define assay conditions allowing the study of F508del CFTR processing and rescue in murine intestinal epithelium in maintenance culture [36], [37]. The assay we have developed can be used to study the trafficking defect of the F508del protein in intact epithelial tissue.

Here we show for the first time that low temperature (26°C) incubation induced the delivery of complex-glycosylated F508del CFTR to the plasma membrane and full functional rescue of CFTR activity in mouse intestinal mucosa. Furthermore, we show that at physiological temperature this rescue action could be mimicked by a variety of proteasome inhibitors (PIs). The latter result contrasts with earlier reports using cell culture models [38], but confirms a later study in a different immortalized cell population [39]. This emphasizes the importance of our approach to examine F508del CFTR rescue in fully differentiated tissue. This preclinical study clearly demonstrates that interfering with the proteolytic activity of the proteasome may restore CFTR function in intact murine epithelium, and suggests that proteasome inhibitors may have therapeutic potential for the treatment of CF [40].

## Materials and Methods

### Materials

William's E medium™ with Glutamax was obtained from Gibco-BRL. Insulin, dexamethasone, the chemicals for the Ussing-chamber measurements forskolin , genistein, carbachol, heat-stable *E. coli* enterotoxin (STa), 8-pCPT-cGMP, bradykinin, ATP and the proteasome inhibitors N-Acetyl-Leu-Leu-Nle-CHO (ALLN), MG-132 and brefeldin A (BFA) were purchased from Sigma. The proteasome inhibitor epoxomicin was obtained from Calbiochem. The ECL detection system was from Pierce.

### Animals

All animal experiments were performed according to the guidelines issued by the Dutch government concerning animal care and work with genetically modified organisms, all breeding of and experiments with normal and mutant animals were performed under permission of the independent institutional animal care and ethics committee of the Erasmus MC (DEC, program numbers OZP 120-54-01; 120-07-05). The F508del CFTR (*Cftr^tm1Eur^*) mice with a mixed background (129× FVB) were backcrossed for more than 13 generations with FVB wild type animals. The animals were fed solid food and acidified water ad libitum. Mortality and weight reduction of mutant animals was below 10–15% compared to normal littermates in this colony. All experiments were performed with 12–18 weeks old, congenic FVB mice carrying the F508del CFTR (Cftr^tm1Eur d/d^) mutation and wild type littermates (Cftr^tm1Eur +/+^) as controls in parallel experiments. *Cftr^tm1cam^* (FVB) knockout mice [35] were used as controls in immunocytochemistry.

### Ussing chamber experiments of murine intestine

Mouse ileum was excised under hypnorm/diazepam anaesthesia. The animals were terminated with an overdose of anaesthetics. The ileum was flushed with ice-cold modified Meyler's solution (105 mM NaCl, 4.7 mM KCl, 1.3 mM CaCl_2_, 1.0 mM MgCl_2_, 20.2 mM NaHCO_3_, 0.4 mM NaH_2_PO_4_, 10 mM HEPES), the muscle-layer was removed and the tissue was mounted in the Ussing- chamber. All experiments were done in duplicate. At the serosal side glucose (10 mM) and indomethacin (10 µM) were added throughout the whole experiment in order to serve as an energy source and to reduce basal Cl^−^ secretion caused by endogenous production of prostaglandins, respectively. After reaching equilibrium, the short-circuit current (Isc) response to the following compounds was recorded, added either mucosally (M) or serosally (S): forskolin (10 µM, S), to provoke cAMP-induced, CFTR-mediated anion secretion; genistein (100 µM, S+M), acting as a potentiator of the F508del CFTR channel [41]; carbachol (200 µM, S), a Ca^2^-linked secretagogue acting mainly through activation of basolateral K^+^ channels and enhancement of the driving force for apical Cl^−^ exit [42]; and D-glucose (10 mM, M), to measure Na^+^-coupled glucose uptake by the SGLT1 transporter in the intestinal villi.

### Tissue incubation

After obtaining the initial Isc recordings (t = 0), the tissue was incubated in gassed (95% O2/5% CO_2_) William's E medium**™** with Glutamax (Gibco-BRL) supplemented with penicillin/streptomycin (100 U/ml), insulin (10 µg/ml) and dexamethasone (20 µg/ml) under the following conditions: 1) Low temperature incubation: 26°C for 4 h–14 h; 2) Incubation with proteasome inhibitors at 37°C for 2 h–6 h: ALLN (20 µM), MG-132 (10 µM), epoxomicin (1 µM), bortezomib (PS314, Velcade™, 8 nM, courtesy University Hospital Innsbruck, Austria); 3) Incubation with brefeldin A (BFA, 20 µg/ml) at 37°C. Each piece of ileum was subjected to serial Ussing-chamber measurements to obtain recordings at different time points. Effects of the compounds were judged by the difference in Isc responses before and after treatment.

### Immunohistochemistry

Tissues were fixed in 4% (wt/vol) para-formaldehyde. Sections (5 µm) were stained with the rodent-specific CFTR antibody R3195 (1∶500) as described elsewhere [6].

### Preparation of tissue samples for Western-blotting

Epithelial cells from treated ileum were isolated by exposing the tissue to vibration (Vibromixer type E1 from Chemap A.G., 50 Hz, amplitude 1.5 mm). Crude microsomal membranes were generated from the isolated enterocytes as described previously [43]. Western-blots were prepared according to standard procedures using the Bio-Rad Miniprotean apparatus system. Murine CFTR was detected with the R3195 (1∶1000) antibody using the ECL detection system (Pierce™). HRP-conjugated secondary antibody was detected by chemoluminescence and the imaged bands (in particular CFTR band C and band B) were visualized on light-sensitive imaging film (Biomax XAR film, Kodak) and quantified using a calibrated GS-800 densitometer (Bio-Rad) and Quantity-one software (version 4.2.1; Bio-Rad).

## Results

### Complete functional rescue of F508del CFTR after low temperature incubation

To test the efficacy of CFTR rescue compounds in murine intestinal biopsies *ex vivo*, functional stability of the tissue under maintenance culture conditions is a major challenge. The *Cftr^tm1eur^* F508del CFTR homozygous mutant mice display a much reduced but distinct residual CFTR mediated Cl^−^ secretion in ileal mucosa under Ussing-chamber conditions [35]. Compared to mice homozygous for the *Cftr^tm1Cam^* knockout allele in the same background (FVB), there is a significant cAMP-induced secretory Cl^−^ current (∼20% of WT littermates) that can therefore be attributed to residual activity of F508del mutant CFTR. However, this response does not survive prolonged incubation at 37°C in glucose-containing minimal salt medium (modified Meyler's solution) for >4 h. A considerable improvement of functional survival could be reached by replacing the Meyler's buffer by William's E-Glutamax medium supplemented with insulin (10 µg/ml) and dexamethasone (20 µg/ml). Ussing-chamber measurements of F508del murine tissue incubated at 37°C in William's E medium showed a gradual decrease of CFTR-dependent ion transport activity of 40% after 8 hours and 60% after 19 hours of incubation (not shown). However, no significant loss of current response was seen after 4–6 h of incubation, indicating that short term testing of CFTR potentiators and correctors in this *ex vivo* assay is feasible.

**Figure 1:**
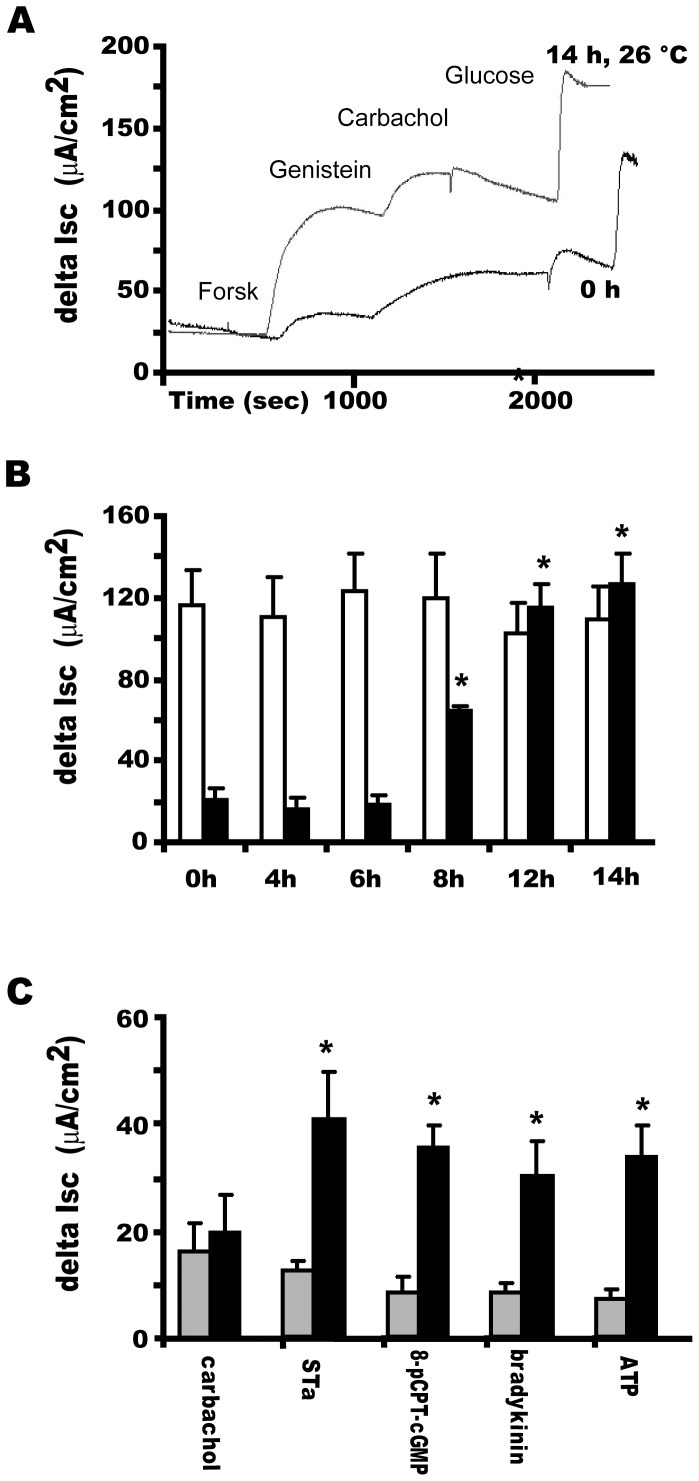
Functional rescue of murine F508del CFTR in mouse ileum after low temperature incubation. Pieces of normal and F508del mutant murine ileal mucosa were incubated for different times at 26°C. The same piece of tissue was subjected to short- circuit current measurements (Isc) at the start of the experiment (t = 0) and following incubation in supplemented William's E medium (4 h–14 h). **A.** Representative tracing showing temperature rescue (14 h at 26°C) of mutant F508del CFTR activity compared to 0 h. **B.** Time course of temperature rescue of ▪: ileum from homozygous F508del-Cftr mice, compared to □: WT ileum. Delta Isc represents the change in short-circuit current from baseline after addition of forskolin *plus* genistein. **C.** Overnight incubation (t = 14 h) of F508del CFTR ileal mucosa at low temperature. Bioelectric characteristics of F508del ileum at t = 0, (□) and after incubation at reduced temperature (26°C, t = 14 h, ▪). Delta Isc represents the change in short-circuit current from baseline after addition of carbachol (100 µM; S), STa (0.1 µM; M), 8-pCPT-cGMP (50 µM; S), bradykinin (2 µM; S), or ATP (100 µM; M+S). N = 4–6 mice, 2 ileal pieces/mouse. Bars represent averages +/− SD (N = 6 mice, 2 samples per mouse. * indicates significantly different from 0 h, P<0.01 by ANOVA).

The most common strategy to rescue the F508del CFTR processing in cell culture experiments is exposure to low temperature (26°C). We investigated the effect of reduced temperature incubation on the forskolin/genistein-stimulated Cl^−^ secretion of freshly excised murine intestinal tissue derived from homozygous F508del mice and their wild type littermates. The tissue was subjected to serial Ussing-chamber measurements under short-circuit current conditions, and incubated at 26°C for different periods. A representative tracing from experiments involving two samples from six mutant and normal mice each, shows temperature rescue of cAMP-dependent Cl^−^ secretion (delta Isc) after 8 h of incubation (Figure 1A). Measurements at different time points first show significant rescue after 8 h, and maximal rescue to wild type levels at 14 h. (Figure 1B) and persisted up to 48 h (data not shown). The Na^+^ -coupled glucose uptake response, which was routinely measured as a CFTR independent indicator of the viability of the tissue, was not significantly altered after the prolonged incubations (N = 8; data not shown). In contrast, the cAMP-provoked Cl-secretion in ileum of wild type mice did not increase further during incubation at the reduced temperature (Figure 1C). This outcome is in line with our previous notion that the CFTR conductance is no longer rate-limiting for trans-epithelial Cl^−^ transport at CFTR protein levels above ∼20% of non-CF controls [42].

After overnight low temperature incubation (14 h) of mutant ileum also the CFTR dependent Cl^−^ secretory response to cGMP-linked secretagogues (heat-stable enterotoxin, STa; 8-pCPT-cGMP), purinergic agonists (ATP), and a non-cholinergic Ca^2+^-linked agonist (bradykinin), were restored to near WT levels (Figure 1C). Unexpectedly, there was no clear improvement of the Ca^2+^ dependent secretory response to carbachol (Figure 1C). However the Isc response to carbachol of WT ileum, but not to any of the other secretagogues, was likewise strongly reduced upon O/N low-temperature incubation (results not shown), suggesting that prolonged *in vitro* incubation results in the loss of carbachol-specific signalling components, e.g. muscarinic receptors.

### Maturation of murine F508del CFTR induced by low temperature incubation

**Figure 2:**
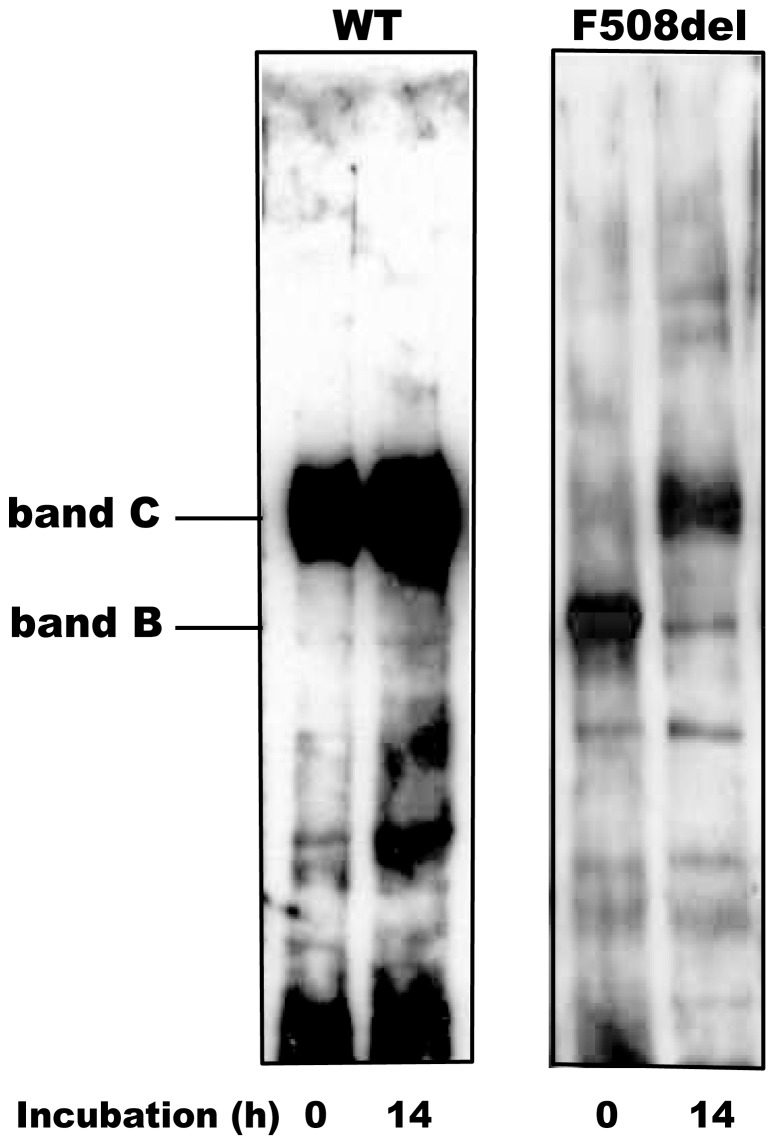
Maturation of F508del protein at low temperature. Pieces of ileal mucosa (∼5 cm) isolated from WT or homozygous F508del CF mice were mounted in large aperture tissue holders and incubated at 26°C for 14 h under carbogen gassing. Subsequently, epithelial cells were harvested by high-frequency vibration and a crude membrane fraction was isolated and subjected to SDS-PAGE (20 µg protein/lane). Wild type (WT) and F508del *Cftr* was detected by Western blotting using the R3195 (1∶1000) antibody. A representative result of four different experiments is shown. Band intensities were estimated by digital scanning (see Methods), showing an increase of F508del CFTR band C to approximately 25% (+/−5 SEM, N = 4) of wild type levels.

Core-glycosylated, immature CFTR (band B) is localized in the endoplasmic reticulum (ER), whereas the complex glycosylated, mature CFTR (band C) is present in compartments distal to the Golgi, in sub-apical compartments and in the apical membrane of epithelial cells. Crude membrane preparations isolated from murine intestinal mucosa contain a mixture of ER and apical membranes, and are suitable to determine the tissue content of both immature (band B) and mature (band C) CFTR protein. As shown in Figure 2, over 98% of the CFTR pool in ileal mucosa from wild type mice consisted of band C, both before (t = 0 h) and after (t = 14 h) low temperature incubation, as determined by digital scanning (see methods) . In contrast, the western blots of crude ileal membranes freshly isolated from homozygous F508del *Cftr* mutant mice showed that more than 95% of the CFTR protein content is band B (Figure 2, t = 0 h). This low band C expression in intestinal membranes from F508del *Cftr* mice was previously reported [6] and recently confirmed in isolated brush border membranes [44]. However, following incubation for 14 h at the reduced temperature a clear shift from band B to band C occurs, indicative for enhanced maturation or reduced degradation of F508del CFTR at this temperature (Figure 2). The level of F508del CFTR band C amounted to approximately 25% of the wild type band C (N = 4 independent experiments), which is sufficient to account for the full restoration of trans-epithelial Cl^−^ secretory currents shown in Figure 1A.

**Figure 3:**
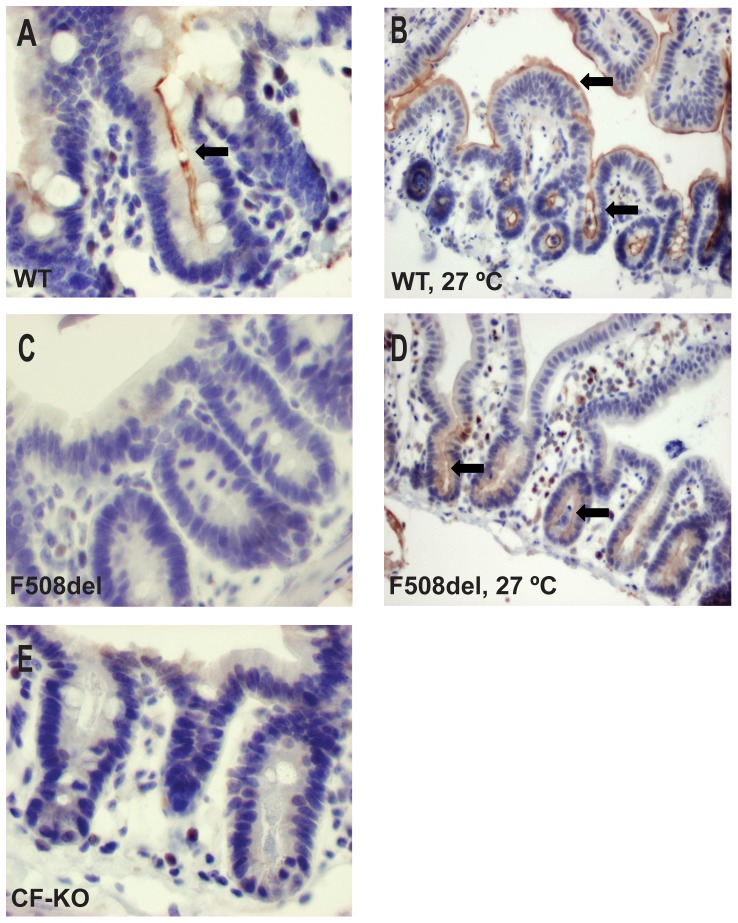
F508del CFTR is detectable by immunostaining after low temperature incubation. Immunohistochemical staining of CFTR in ileum of wild type (WT, A and B) and homozygous F508del (F508del, C and D) mice prior to (A,C) and following low temperature incubation (t = 14 h, B, D). F508del CFTR is only detectable after low temperature incubation. Ileal sections of homozygous *Cftr^tm1Cam^* (FVB) knockout mice were used as negatieve controls for the immunostaining (CF- KO, E). Magnifications: A, C, E: 400× and B, D: 200×.

Paraffin sections derived from wild type ileum prior to low-temperature incubation showed apical CFTR protein staining in the intestinal crypts and a less prominent staining in the apical membrane of the villi (Figure 3A). After 14 h incubation at 26°C an increase in apical CFTR protein expression was observed in both the epithelial crypt and villus compartment (Figure 3B). Prior to low-temperature rescue, the F508del CFTR protein expression remained below the detection limit for immunostaining (Figure 3C). However, after incubation at low temperature (t = 14 h), surface expression of the F508del protein at the apical border of intestinal crypts was readily detected, albeit at a substantially lower level than in wild type mice (Figure 3D). Tissue derived from *Cftr^tm1Cam^* CFTR knockout mice co-incubated on the same slide was negative in all cases, indicative for the specificity of the antibody (Figure 3E). These intestinal data confirm and extend previous data from cultured gall bladder cells showing that murine F508del CFTR, similar to the human ortholog, displays a temperature sensitive processing defect [6]. Moreover, we establish muscle-stripped ileal mucosa from *Cftr^tm1Eur^* mutant mice as a platform to study *ex vivo* F508del correction in the context of fully differentiated epithelium.

### Proteasome inhibitors restore chloride secretion in F508del intestine

Initially, pulse-chase experiments of CFTR transfected HEK and CHO cells showed the accumulation of detergent-insoluble, multi-ubiquitinylated CFTR molecules with no detectable increase of folded CFTR after proteasome inhibition [38], [45]. However, more recent studies by Gentzsch et al. on the endocytic trafficking of wild type and F508del CFTR in BHK cells demonstrated that proteasome inhibitors can stabilize low temperature rescued mutant protein by preventing lysosomal degradation and promoting recycling of endocytosed CFTR back to the plasma membrane [46]. Comparable results were presented by Vij et al in a similar immortalized cell line [39].

**Figure 4:**
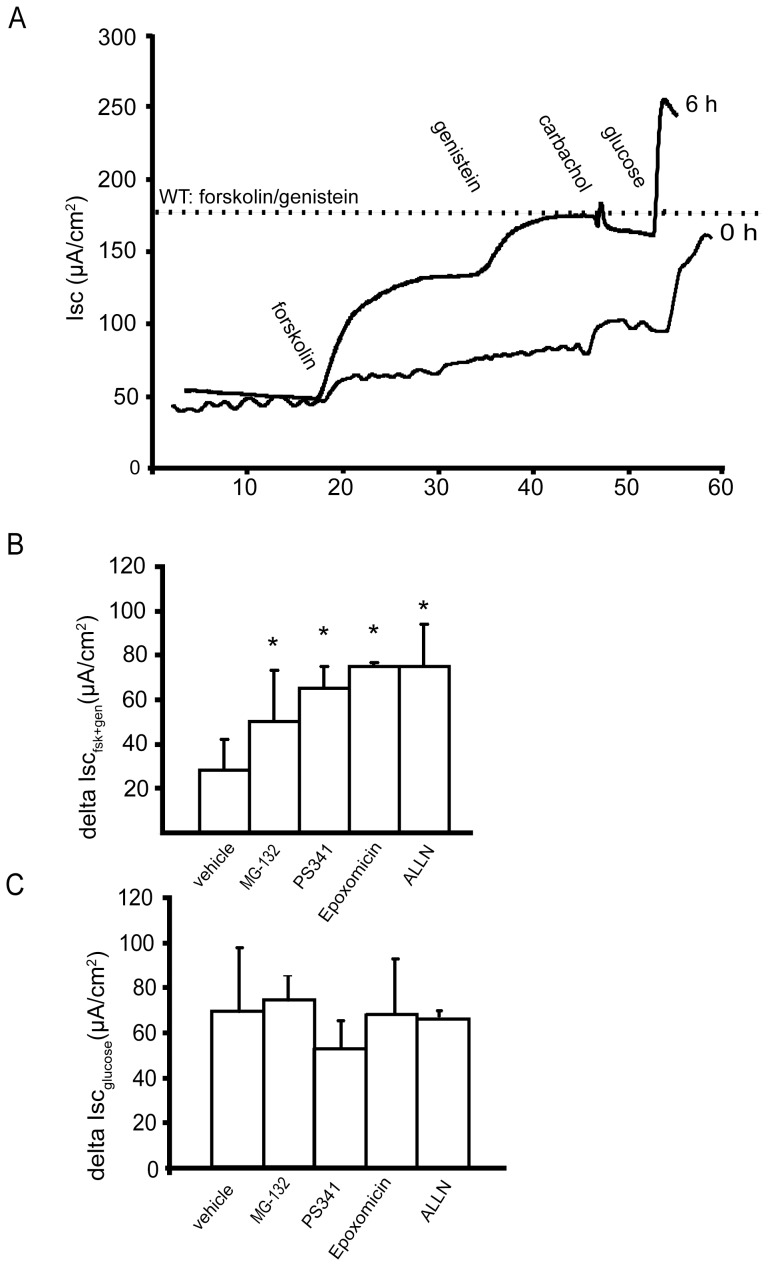
Functional rescue of F508del CFTR in native ileal mucosa after treatment with proteasome inhibitors. **A.** Representative Isc tracing of ileum from homozygous mutant F508del mice in the Ussing-chamber before (t = 0 h) and after treatment with the proteasome inhibitor ALLN (t = 6 h). Residual forskolin and genistein mediated chloride secretion at the start of the experiment (t = 0 h) was observed in all experiments. The dotted line indicates the average short circuit current level following forskolin/genistein addition for WT ileum (N = 24). ALLN treatment resulted in complete correction of CFTR activity compared to wild type. **B.** Ileal mucosa from F508del/F508del mutant mice was mounted in Ussing- chamber holders and incubated under carbogen gassing in William's E medium for 4 h at 37°C in the presence of vehicle (DMSO, 0.1%), MG-132 (10 µM), epoxomicin (1 µM ), PS341 (30 nM) or ALNN (20 µM). Subsequently the Isc response to forskolin *plus* genistein was measured in Ussing chambers. **C.** At the end of each measurement the viability of each sample was tested by addition of 10 mM glucose to the mucosal bath. The average glucose response after treatment with the various proteasome inhibitors is not significantly different from the vehicle control. All experiments were performed with N = 4 mice, two samples per mouse. Averages plus or minus SD are shown, statistical analysis was by one way ANOVA with Tukey post hoc test for multiple averages, *: indicates significantly different P<0.05 from vehicle).

These apparently contradictory studies prompted us to use our ex vivo intestinal model for testing the ability of proteasome inhibitors to rescue F508del CFTR function in native intestinal epithelium. Muscle stripped ileum from homozygous mutant *Cftr^tm1Eur^* mice was incubated at 37°C for two to six hours in supplemented William's E medium containing different proteasome inhibitors (PIs), or vehicle alone as control. The forskolin *plus* genistein induced short-circuit current (Isc) response was determined at t = 0 h and t = 4–6 h. As shown in Figure 4A, incubation of F508del mutant ileum with the PI ALLN for 6 h resulted in a full restoration of transepithelial anion secretion, up to wild type values. Recovery by this PI at 37°C was two times faster than low temperature rescue (Figure 1B).

All four different PIs tested in this assay significantly increased the forskolin/genistein-stimulated CFTR mediated chloride secretion, whereas incubation with vehicle only had no effect (Figure 4B). After 4 h incubation at 37°C MG-132 caused an approximately twofold gain in anion current. The 20S proteasome specific PI's epoxy-ketone epoxomicin and ALLN allowed correction to ∼60% of wild type responses at this time point. Importantly, the clinically relevant PI compound PS341 (bortezomib, Velcade™) showed a similar gain in anion secretion.

No significant change in the glucose response was observed in all tissues tested indicating that inhibition of the proteasome had no effect on tissue viability in our assay (Figure 4C).

### Maturation of murine F508del CFTR is enhanced by proteasome inhibitors

**Figure 5:**
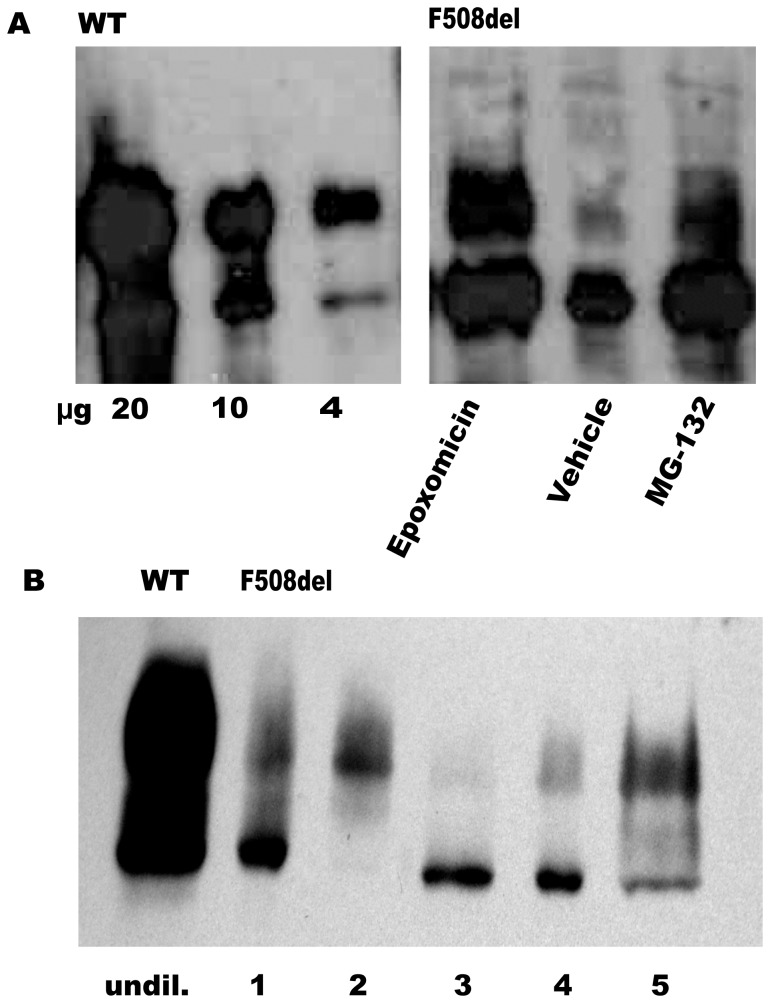
Improved CFTR maturation after treatment with proteasome inhibitors. Pieces of ileal mucosa (∼5 cm) isolated from homozygous F508del CF mice were mounted in large aperture tissue holders and exposed for 4 h (panel A) or 2–6 h (panel B) at 37°C to various PIs (see legend Fig. 4). Subsequently, epithelial cells were harvested by high-frequency vibration and a crude membrane fraction was isolated and subjected to western blot analysis of CFTR as described in Methods. Autoradiographs of 3 independent experiments were scanned, representative figures are shown here. Slots were loaded with equal amounts of protein to facilitate comparison. **Panel A, left**: dilution series of crude membranes derived from wild type mice (WT; 20, 10 and 4 µg protein/lane) used to calibrate the intensity of the wt CFTR signal. **Panel A, right**: crude membranes (20 µg protein) derived from homozygous F508del mice treated for 4 h with epoxomicin (1 µM); vehicle (0.1%DMSO), or MG-132 (10 µM). Average band C intensity increased from 5% (vehicle) to 20% (epoxomicin) and 15% (MG-132) of WT. Band B intensity increased twofold after epoxomicin and MG-132, compared to vehicle. **Panel B:** Crude membranes from wild type mice (WT; 20 µg protein/lane); F508del: crude membranes derived from homozygous F508del mice, untreated (lane 1); following low temperature incubation (26°C, t = 14 h) (lane 2); after treatment at 37°C with ALLN (20 µM) for 2 h (lane 3); 4 h (lane 4); and 6 h (lane 5). Band C/Band B ratio increased from 0.08 (2 h) to 0,36 (4 h) and 4,8 (6 hr).

The increase of cAMP-dependent chloride secretion in PI-treated tissue was accompanied by the increased appearance of mature, complex glycosylated (band C) CFTR in western blots. Scanning of the gels showed that compared to carrier treated F508 CFTR, epoxomicin and MG-132 increased the band B intensity twofold and the band C intensity three to fourfold (Figure 5 A). Incubation with ALLN for 2, 4 and 6 hrs likewise resulted in a time-dependent increase of band C, and a significant increase in the band C/band B ratio from 0.08 to 0.36 and 4.8 respectively (Figure 5B). As shown in Figure 4A and B, this partial rescue of F508del CFTR band C after 6 hr and 4 hr ALLN is sufficient for near hundred and sixty percent of normal forskolin response respectively. As inferred from a calibration plot of WT-CFTR (Figure 5A), the intensity of the F508del CFTR band C after 6 hours of ALLN treatment was about 20% of the value found in epithelial membranes from WT ileum. In contrast to epoxomicin and MG-132, ALLN treatment did not result in a significant gain in the amount of CFTR band B (Fig. 5B), suggesting that ALLN is a comparatively less effective inhibitor of proteasome function. Importantly, the increase in band B levels following epoxomicin or MG-132 treatment did not result in an enhanced CFTR function compared to ALLN (Figure 4), arguing against unconventional trafficking of core-glycosylated CFTR to the luminal surface under these conditions [47].

### Proteasome inhibitors increase apical F508del CFTR antigen in ileal enterocytes

**Figure 6:**
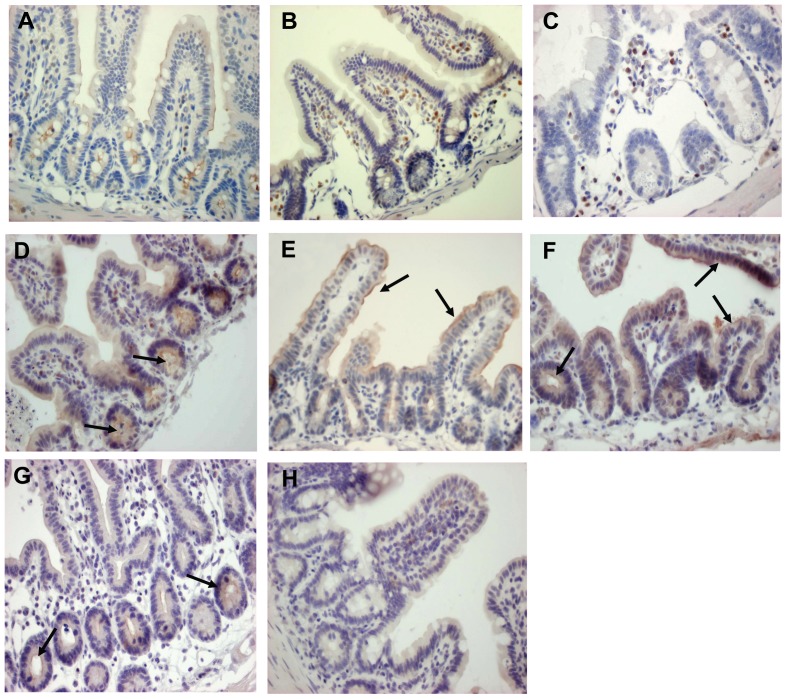
Effect of proteasome inhibitors on CFTR immunostaining. Histochemical staining of CFTR in ileum from FVB wild type mice (A); ileum from untreated homozygous *Cftr^tm1Eur^* F508del mice (B); ileum from homozygous *Cftr^tm1Cam^* knockout mice (C); PI-treated ileum from homozygous *Cftr^tm1Eur^* F508del mice (D–G); ileum was treated for 4 h at 37°C with 1 µM epoxomicin (D), 10 µM MG-132 (E), 20 µM ALLN (F), or 30 nM PS341 (G). Panel H: Vehicle-treated (0.1% DMSO) ileum from homozygous *Cftr^tm1Eur^* (FVB) F508del mice. Magnifications: 200×.

Intestinal mucosa exposed to PIs *in vitro* and analysed in the Ussing-chamber were fixed in 4% para-formaldehyde overnight, embedded in paraffin and stained with the R3195 anti-CFTR antibody [6]. Immunostaining of CFTR protein in untreated or vehicle-treated F508del intestine remained below detection level (Figure 6B, H), confirming present (Figure 3) and previous results [35]. However, following 4 h exposure to epoxomicin (Figure 6D) or PS341 (Figure 6G), apical staining of F508del CFTR was clearly detectable in the crypt compartment, the site of CFTR biogenesis in the intestinal epithelium. After 4 h exposure to MG-132 (Figure 6 E) or ALLN (Figure 6 F), apical localization of murine F508del CFTR protein was observed, also in the lower villus region.

### Rescue of F508del CFTR ion transport function by ALLN in the intestine occurs at the post-Golgi level

**Figure 7:**
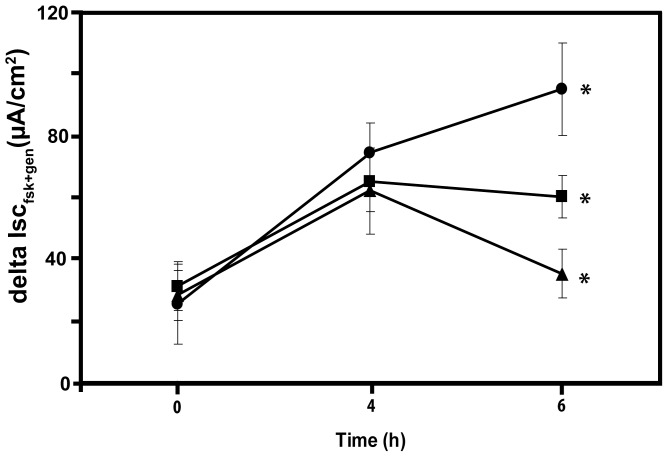
ALLN rescue of F508del CFTR function persists in the presence of brefeldin. Short-circuit current responses to forskolin *plus* genistein were measured in stripped ileum from homozygous mutant *Cftr^tm1eur^* mice (t = 0 h), and again after 4 h of incubation with ALLN as described in the legend of Fig. 4 (4 h). The tissues were then incubated for an additional 2 h in the presence of ALLN alone (closed circles), ALLN plus BFA (closed squares) or BFA alone (closed triangles). At t = 6 h the tissues were again analysed in the Ussing-chamber. Delta Isc represents the change in short-circuit current from baseline after addition of forskolin and genistein (average ±SEM of 3 experiments). All three averages at 6 hrs are significantly different (P<0.05 by one way ANOVA, with Tukey post hoc test for multiple averages).

The rescue of F508del CFTR mediated anion secretion by PIs could take place at the level of the ER, e.g. by inhibition of ERAD [48], or in post-ER compartments, e.g. by stabilizing the mutated protein in the apical membrane [14]. In an attempt to discriminate between both possibilities, we measured the functional rescue of F508del CFTR in the presence of brefeldin A (BFA). BFA blocks protein transfer from the ER to the Golgi system but does not affect the trans-Golgi network (TGN), and therefore prevents the transfer of F508del CFTR from the ER but not from the trans Golgi system to the apical membrane. We first rescued F508del CFTR in mouse ileum by incubation with ALLN for 4 h at 37°C, resulting in a substantial increase of CFTR dependent short-circuit current (Figure 7). Subsequently, the tissue was exposed for 2 additional hours to ALLN alone, BFA alone, or BFA *plus* ALLN, and the effect on transepithelial anion secretion was measured in the Ussing-chamber. In the absence of BFA, ALLN further enhanced cAMP-stimulated anion secretion up to the level observed in WT ileum, either by preventing F508del CFTR degradation in the ER, or by stabilizing a pool of F508del CFTR in the apical membrane. Inhibition of Golgi to apical membrane traffic by BFA alone resulted in a rapid return of ALLN-rescued anion secretion back to pre-ALLN levels. This rapid loss of the rescued F508del CFTR in the apical compartment in the presence of BFA could be prevented at least partially by ALLN. This is consistent with the existence of an ALLN-sensitive post-Golgi degradation pathway for murine F508del CFTR in intestinal cells.

In summary, these data show that inhibition of the proteasome leads to a substantial improvement of murine F508del CFTR protein expression, maturation and function and demonstrate that the ubiquitin-proteasome pathway is involved in the degradation of murine F508del CFTR in native epithelium.

## Discussion

Here we describe an *ex vivo* assay applied to muscle-stripped mouse intestinal mucosa that can be used to test experimental therapies aimed at improving the function of F508del CFTR. First we demonstrate that complete functional rescue of the murine F508del CFTR activity can be achieved in this model by incubation of the tissue at low temperature (Figure 1). The functional rescue was accompanied by partial rescue of F508del CFTR protein maturation, as shown by western blotting (Figure 2) and immuno-histochemistry (Figure 3). As a demonstration of an effective pharmaceutical approach we show here that incubation of mutant ileal mucosa with four different proteasome inhibitors at physiological temperature (ALLN, epoxomicin, MG-132, PS341/bortezomib) also results in substantial rescue of the mature F508del CFTR protein and of its anion transport function (Figures 4–7). As expected from the exceptionally high turnover rate of small intestinal epithelium in comparison with other epithelial tissues, 2–3 days in mice and 5 days in humans [49], biosynthesis of CFTR in the intestinal crypts must be relatively fast, to explain the kinetics of F508del CFTR rescue by low temperature (maximal at 8 h) or in the presence of PIs (maximal at 4 h) found in this study.

Our data indicate that degradation of misfolded CFTR by the proteasome, or by a process indirectly linked to proteasome function, is the major mechanism accounting for the instability of murine F508del CFTR in native intestinal epithelium. Furthermore, the effect of brefeldin A on ALLN induced rescue (Figure 7) suggests that PI-sensitive degradation is not limited to the ER, but also occurs in a post ER compartment, most plausibly involving the peripheral protein quality control system [14].

Previous studies of CFTR degradation have been performed mainly in immortalised human cell culture models. ER associated degradation (ERAD) of F508del CFTR was found to involve the ubiquitin-proteasome pathway [50]. Additionally, the activity of an ATP independent-pathway has been suggested that is responsible for the degradation of misfolded F508del CFTR [45]. Studies of proteasome inhibitors in these model systems did show stabilisation of band B but failed to demonstrate improved maturation to band C [38], [45].

Our data extend these reports by showing that in the context of differentiated mouse cells efficient functional correction by PI's of murine F508del CFTR does occur. This raises the question whether in differentiated human cells *in situ* this can be observed as well, and whether PI's can contribute to the treatment of CF in humans.

Here, we clearly demonstrate in native mouse intestine that proteasome inhibitors are capable of preventing degradation of murine F508del CFTR in the murine enterocyte, allowing the accumulation of fully mature F508del CFTR and partial or full restoration of transepithelial chloride secretion. Furthermore, our data obtained with brefeldin A (Figure 7) suggest that murine F508del CFTR rescue by PI's occurs at least in part at the post-Golgi level, presumably involving stabilization of the surface pool of F508del CFTR. Such a rescue mechanism is expected to be more prominent in species and tissues showing partial processing of F508del CFTR, as demonstrated here for the mouse intestine by the occurrence of a considerable F508del CFTR dependent trans-epithelial anion secretion (∼20% of wild type) and of mature F508del-CFTR protein on western blots (band C; ∼5% of WT; Fig. 2, 5) in the absence of any treatment.

### Mechanisms of PI induced F508del CFTR rescue

Recent studies have shown that F508del CFTR folding efficiency is highly sensitive to secondary mutations in the CFTR sequence [51], [52], and further dependent on the multiple interactions with the large number of proteins that constitute the folding and quality control system of the cell (‘The Cloud’) [10]. It is no surprise therefore that F508del CFTR folding efficiency depends both on the cellular context and the CFTR sequence [53]. This likely also applies to other critical stages of the CFTR life cycle: transfer through the Golgi, glycosylation, insertion and recycling at the plasma membrane. At each of these levels PI's potentially have an effect on F508del CFTR turnover either through a direct interaction with ubiquitin tagged CFTR, or indirectly though affecting the turnover of a key factor in the proteostasis machinery. It is conceivable, therefore, that PI's will have a different effect on human airway or intestinal cells *in situ* than in immortalised cells in culture. A PI induced accumulation of the CFTR precursor (band B) as observed in cultured human cells, was also observed with epoxomicin and MG-132 in our system (figure 5A). This may by itself not be sufficient for functional correction, but is likely show synergy with a suitable folding corrector and activator. Moreover, is seems likely that stabilisation of corrected human F508del CFTR at the apical membrane also occurs. Together this may increase the level of correction to levels required for clinical benefit. Indeed, Gentzsch et al.showed, most recently in a model of differentiated human airway epithelial cells that surface derived normal and F508del CFTR is recycled through endocytosis [46], [54]. The mutant form is recycled less efficiently, and is retained and subsequently degraded in sub-apical vesicles. Moreover, the internalization and lysosomal breakdown of this surface pool of mutant CFTR could be largely prevented by incubation with PI's [54]. In a related study it was shown by Lukacs et al. that improperly folded domains in F508del CFTR are recognised at the plasma membrane by the Hsc70/Hsp90 chaperone system, which allows CHIP mediated ubiquitination, internalisation and finally degradation of mutant CFTR in the lysosomes [14]. The mechanism by which PIs cause retention of mutant CFTR at the cell surface is still unclear but may involve inhibition of the ubiquination step initiating the internalization of F508del CFTR at the apical membrane, possibly by depletion of free ubiquitin [54].

Recent data by Maiuri et al. redefine CF as a deficiency of autophagy. They show that abnormal trafficking of human F508del CFTR in cell culture and murine F508del CFTR can be effectively rescued by ROS scavengers and inhibition of transglutaminase [55], which increases the efficacy of CFTR activators [56]. Future studies will be required to establish the effect of PIs on this process, which would offer an alternative explanation for PI induced rescue of mutant F508del CFTR CFTR.

A further alternative explanation for the functional rescue of F508del CFTR in native intestinal epithelium was also considered, i.e. PI stimulation of ER stress and of the unfolded protein response (UPR), possibly resulting in cell surface trafficking of core-glycosylated CFTR through an unconventional pathway [47]. While we can not completely rule out this explanation, several findings argue against the operation of this mechanism under our conditions. First, ALLN has no apparent effect on the steady-state level of F508del CFTR band B (Figure 5B), whereas functional rescue was observed (Figure 4B). Further, all PI's tested increase the level of F508del CFTR band C, in clear contrast to the absence of such effects under conditions of ER stress [47]. Finally, a twofold increase of band B observed following exposure to the PI inhibitors Epoxomicin and MG-132 (Figure 5 A) was not paralleled by an enhanced functional rescue of F508del-CFTR in comparison with ALLN treatment (Figure 4B).

Additional experiments including a more detailed analysis of PI effects on the peripheral quality control system for mutant-CFTR in native mouse epithelium, and studies of F508del-CFTR rescue by PIs in native human intestinal epithelium (e.g. rectal biopsies or organoids) are needed to confirm our concept and to explore the applicability of PIs for the rescue of F508del-CFTR processing and function in CF patients.

### Anti-inflammatory action of proteasome inhibitors

Vij et al. recently showed that nano-particle mediated intranasal delivery of the proteasome inhibitor Velcade/Bortezomib reduced the LPS induced inflammatory response in CFTR KO mice [57]. This was attributed to inhibition of the NFκB pathway, though a reduction of IκBα inactivation. Vij et al. have previously provided evidence that CFTR deficiency results in activation of the NFκB pathway [58], [59]. These studies show the feasibility and efficacy of PS341 (Velcade/Bortezomib) delivery *in vivo*, and combined with our data suggest that a therapeutic effect of proteasome inhibitors in CF pathology can be expected at two interrelated levels, first functional rescue of F508del CFTR processing (this paper, [39]) and further reduction of NFκB activity [40]. Further studies are required to establish whether the effect of PI's on F508del CFTR folding and trafficking may be due to a PI induced reduction of NFκB, which may have an effect on the ER and peripheral QC system.

### Towards clinical application of proteasome inhibitors in CF

Proteasome inhibitors interfere with multiple cellular processes. Therefore, their therapeutic usefulness for the treatment of CF might be questioned. However, the recent development of peptide boronates made the clinical use of proteasome inhibitors feasible [60]. Preclinical studies *in vitro* and *in vivo* using the proteasome inhibitor PS341 (Bortezomib, Velcade™) demonstrated its anti- tumor activity in models for hematological malignancies and solid tumors [61]. PS341 can be used effectively at low concentrations, and clinical trials conducted in patients with refractory hematological malignancies reported antitumor activity with manageable treatment related problems [62], [63], [64]. Patients with advanced solid tumors also benefit from PS341-based combination therapies [65]. More recently, promising results were reported in an animal model with MLN9708, a molecular variant of PS341 with better bioavailability [66], [67].

Our data show that PS431 treatment increased the cAMP dependent chloride secretion in F508del murine intestine (Figure 4B), and a distinct increase in apical CFTR antigen (Figure 6G), with no significant effect on glucose response (Figure 4C) or tissue morphology (Figure 6G). Further development of proteasome inhibitors for long term clinical applications [68], is therefore not only a priority in the field of oncology but also in the field of CF.

Current reports highlight the cell type specific differences in response to pharmaceutical intervention at the level of F508del- CFTR processing. Furthermore the response to correctors varies considerably between cell pools from different donors [29], [34]. Previously, variations of clinical phenotype in patients homozygous for F508del, were shown to correlate with differences in residual F508del CFTR activity [69], [70]. Together, this suggests that in the F508del patient population significant differences in the kinetics of processing and recycling of CFTR can occur. It seems likely, therefore that for clinical benefit in specific F508del CF patients we need to define an optimal combination of agonists, correctors, potentiators and possibly proteasome inhibitors, which act on different levels of the CFTR processing and recycling machinery [10], [51], [52].
